# Dr. Walker, on the Vaccine Areola

**Published:** 1808-10

**Authors:** John Walker

**Affiliations:** Salisbury Court


### *To the Editors of the Medical and Phyfical Journal.*

GENTLEMEN,

I Have not been able to conceive the cause or origin of the cellular structure of the Vaccine Pock. Can the application of an active virus excite, or set up, a creative action in the system, so assailed, or attacked? Dr. Jenner says, "Nature appears to have no more difficulty in forming minute glands, among the vascular parts of the body, than she has in forming blood-vessels, and millions of these can be called into existence, when inflammation is excited, in a few hours."

Mr. Dunning says, "If the vaccine vesicle be a congeries of cells insulated with respect to each other, must we not consider this constitution of it as a wise and beautiful œconomy of Nature for preserving its precious contents from casualty and exposure." He says, moreover, "This undoubtedly is the inference which Dr. Jenner draws, and wishes should be drawn from it."

O Doctor! Doctor! Mortalium felicissime! non solum beneficia certa naturæ hominibus libenter dedisti; sed etiam consilia ejus, satis inexplicabilia, demonstras, et (bone sacerdos!) fidem exigis: et nonnullos habes fidelissimos.

From neither of these statements, romantic or religious, have I been able to form any conception of the cause or origin of the organized structure. Dr. Willan says, "the vesicle consists internally of numerous little cells filled with clear lymph, and communicating with each other. These are, perhaps, only a portion of the cellular membrane, *(tissu-cellulaire)* distended by the effusion of lymph. Most other vesicular eruptions consist of simple vesicles." On casting my eye on this explanation, I was immediately struck with the recollection of the bewildered maid in the mask of Comus. 'It was a sable cloud turned out its silvery lining on the night.' It is cellular membrane, the *rete mucosum*, which unfolds itself, and receives the vaccine ichor as produced in the chemico-animal process, set up, under the cuticle, and which burgeons outward, and presses inward, as the pock advances to its full dimensions.

When it has acquired these, does it act as an extraneous body irritating and inflaming the surrounding muscle? does absorption then take place in part from such excitement; and is the whole system threatened to be overwhelmed with the ingress of a deleterious virus? Does the injury threatened, the violence done, by the immediate absorption absorption of virus, all round the pock, cause the evident, the palpable, the local, morbid, affection, the distinct areola, the erythematous induration, to take place, and thus to form a sort of barrier against the full ingress of a deadly poison? If a hurtful and dangerous ingress be thus prevented, there is, notwithstanding, a general effect produced on the skin, as is evident from the circumstance of its being *now* most rapidly affected by the insertion of vaccine virus into any part of it, as well as from the *hereafter insusceptibility* both of the vaccine and variolous infection. It is remarkable, that in some of those subjects who have already had the small-pox, and in whom there seems to be an insusceptibility of this very great and local effect, this perfectly characteristic erythematous induration, or distinct areola, at the place of insition, there is an evident early susceptibility of a very general effect in the system. Of this I felt very sensible in my own person in less than a week after inoculating myself on the Atlantic, in 1800; so much so, that I should have had the weakness to have been under considerable alarm, as I had not yet met with a similar case; but, just at that time, we fell in with the gun-boats of Algesiras, in our approach to Gibraltar, and received such salutes, (projections of shot and shells, which passed over us or fell short) both from them and the Spanish forts ashore, as caused the apprehension, of a danger that was only imaginary, to give place to that of one which seemed seriously real. I have known several to be considerably affected by such experiments, and by accidental inoculations of the cow-pock, remarking, at the time, that if the variolous inoculation had been found to produce so great an effect, after vaccination, this would never have been adopted. In such subjects, I have frequently, distinctly, noticed the course of the absorbents, along a whole limb, by the inflammation, a thing unusual in perfect vaccination. Such appearance, perhaps, renders the notion, however fanciful, at least plausible; that the erythematous induration, at the pock, may prevent a hurtful, uninterrupted, ingress of a deleterious virus into the system, which has not yet lost its original susceptibility; while that skin, which has been already guarded, by the small-pox, or a previous inoculation, is not now sufficiently irritable, or excitable, in any part of it, to have such distinct and circumscribing induration produced, by the application of the virus, however active.

Respectfully,

JOHN WALKER.

*Salisbury Court, 18, ix, 1808.*

